# Increased migration of antigen presenting cells to newly-formed lymphatic vessels in transplanted kidneys by glycol-split heparin

**DOI:** 10.1371/journal.pone.0180206

**Published:** 2017-06-30

**Authors:** Ditmer T. Talsma, Kirankumar Katta, Miriam Boersema, Saritha Adepu, Annamaria Naggi, Giangiacomo Torri, Coen Stegeman, Gerjan Navis, Harry van Goor, Jan-Luuk Hillebrands, Saleh Yazdani, Jacob van den Born

**Affiliations:** 1 Department of Nephrology, University Medical Centre Groningen, Groningen, Netherlands; 2 Department of Pathology and Medical Biology, University Medical Centre Groningen, Groningen, Netherlands; 3 Ronzoni Institute, Milano, Italy; UCL Institute of Child Health, UNITED KINGDOM

## Abstract

**Background:**

Chronic renal transplant dysfunction is characterized by loss of renal function and tissue remodeling, including chronic inflammation and lymph vessel formation. Proteoglycans are known for their chemokine presenting capacity. We hypothesize that interruption of the lymphatic chemokine–proteoglycan interaction interferes with the lymphatic outflow of leukocytes from the renal graft and might decrease the anti-graft allo-immune response.

**Methods:**

In a rat renal chronic transplant dysfunction model (female Dark-Agouti to male Wistar Furth), chemokines were profiled by qRT-PCR in microdissected tubulo-interstitial tissue. Disruption of lymphatic chemokine–proteoglycan interaction was studied by (non-anticoagulant) heparin-derived polysaccharides in vitro and in renal allografts. The renal allograft function was assessed by rise in plasma creatinine and urea.

**Results:**

Within newly-formed lymph vessels of transplanted kidneys, numerous CD45^+^ leukocytes were found, mainly MHCII^+^, ED-1^-^, IDO^-^, HIS14^-^, CD103^-^ antigen presenting cells, most likely representing a subset of dendritic cells. Treatment of transplanted rats with regular heparin and two different (non-)anticoagulant heparin derivatives revealed worsening of kidney function only in the glycol-split heparin treated group despite a two-fold reduction of tubulo-interstitial leukocytes (p<0.02). Quantitative digital image analysis however revealed increased numbers of intra-lymphatic antigen-presenting cells only in the glycol-split heparin group (p<0.01). The number of intra-lymphatic leukocytes significantly correlates with plasma creatinine and urea, and inversely with creatinine clearance.

**Conclusions:**

Treatment of transplanted rats with glycol-split heparin significantly increases the number of intra-lymphatic antigen presenting cells, by increased renal diffusion of lymphatic chemokines, thereby increasing the activation and recruitment of antigen presenting cells towards the lymph vessel. This effect is unwanted in the transplantation setting, but might be advantageous in e.g., dendritic cell vaccination.

## Introduction

Chronic transplant dysfunction (CTD) is characterized by decline in kidney allograft function over time and is related to progressive tissue remodeling in the transplanted kidney. Histologically, CTD is characterized by chronic lesions such as interstitial fibrosis and tubular atrophy, transplant vasculopathy, focal segmental glomerulosclerosis and lymphangiogenesis [1–3]. Lymphangiogenesis is the growth of new lymph vessel via sprouting from existing lymph vessels or by trans-differentiation of monocytes/macrophages into lymphatic endothelial cells, processes which are often initiated by tissue inflammation [4]. A common hypothesis nowadays is that inflammation and lymphangiogenesis have mutual interactions. In the kidney, inflammation promotes lymph angiogenesis by stimulating the production of lymphangiogenic factors like vascular endothelial growth factor C (VEGF-C) by activated tubular epithelial cells and macrophages [5,6], while lymph vessels can produce pro-inflammatory cytokines and form the major exit route for leukocytes. MHC class II expressing antigen presenting cells (APCs), mainly dendritic cells (DCs), are believed to be the leukocyte subtype exiting the interstitium via lymph vessel in CTD and promote allorecognition and alloreactivity.

In transplantation, the exact role of lymphangiogenesis is not yet clear. Although a beneficial hyaluronan draining effect of newly formed lymph vessels in lung transplants has recently been discovered [7]. We and others showed that lymphangiogenesis is associated with poor transplant prognosis [8,9], which might be related to an increased migration of DCs to lymph nodes in transplanted organs suffering from lymphangiogenesis. Increased DC migration in turn leads to increased antigen presentation in lymph nodes resulting in a stronger T and B lymphocyte-mediated allo-immune response [6]. Efforts have been undertaken to block lymph vessel formation in experimental transplantation models and has been shown to increase graft survival [10]. However in the kidney, lymph vessel blockade might lead to renal edema, which has already been shown in mTOR inhibitors like rapamycin and is thought to be due to the inhibition of lymphangiogenesis [11]. Therefore, instead of focusing on the inhibition of lymphangiogenesis, reduction of renal interstitial leukocyte in- and efflux might be a way to improve renal function and graft survival.

Most research on the mechanism and function of APCs has been done in DCs, which are believed to be the most important APC. In DCs, pathogenic or non-self-antigen uptake causes maturation and subsequent C-C chemokine receptor 7 (CCR7) expression on the cell surface. Chemokine (C-C motif) ligand (CCL) 19 and CCL21 are ligands for CCR7 and both have been shown to stimulate DC migration [12,13]. CCL21 is produced by lymphatic endothelial cells and stored intracellularly. Upon release CCL21 and CCL19 bind to heparan sulfate proteoglycans (HSPGs) present in the basement membrane of lymph vessels and form a short chemotactic gradient around the lymph vessel [14,15]. The sulfation pattern of heparan sulfate (HS) can influence its capability to bind CCL19 and CCL21 as shown by Yin and colleagues [16].

Besides CCL21 and 19, the lymph endothelial cells produce other chemokines like CX3CL1 and CXCL12 which are also believed to attract DCs via a chemotactic gradient. CXCL12 probably via HS binding, but since CX3CL1 is a non-HS binding chemokine it is believed to form a fluid phase gradient [17,18]. It is currently believed that the CX3CL1 and CXCL12 chemotactic gradient causes DC migration towards the lymph vessel, where high concentrations of CCL21 cause integrin activation and subsequent ICAM-1-mediated transmigration through lymphatic endothelium [18]. CC-chemokines like CCL2 and CCL5, involved in HS mediated DC influx from the blood, might also play a role in DC migration towards the lymphatic system since it has been shown that these chemokines are upregulated in lymphatic endothelial cells in inflammatory conditions [19].

It has been recognized already for some time that proteoglycans play a pivotal role in the inflammatory process. Therefore strategies have been developed to use the proteoglycan chemokine interaction as a target for therapy. In kidney transplantation it has been shown that treatment with multiple low molecular weight heparins reduce signs of progressive renal failure in experimental renal transplantation [20,21]. In these studies the authors showed that low molecular weight (LMW) heparin reduces renal monocyte, T-cell and major histocompatibility complex II positive (MHCII^+^) infiltration. However none of these studies reported on the effect of heparinoids on the migration of leukocytes towards the lymph vessels. Furthermore, these studies were done with LWM heparin with anti-coagulant properties besides anti-inflammatory properties. Anti-coagulant heparin has been shown to result in an increased risk of hemorrhagic complications in patients early after transplantation [22]. This shows that the anti-coagulant function of heparin is a major burden for the application of heparin in anti-inflammatory treatment. However in experimental renal ischemia-reperfusion injury, it has been demonstrated that treatment with a synthetic and non-anticoagulant heparin pentasaccharide reduced inflammation and neutrophil accumulation [23,24]. This shows that non anti-coagulant heparins might still exert anti-inflammatory effects despite losing their anti-coagulant function. Therefore in this study we used, besides unfractionated heparin, two clinically more favorable non anti-coagulant heparin derivatives, to evaluate the potency of these heparins to interrupt the proteoglycan—chemokine interaction.

In this study we hypothesized that newly-formed lymph vessels in renal grafts are actively involved in leukocyte migration from the graft towards the draining lymph node in order to build an alloresponse. Secondly we hypothesized that interruption of chemokine–proteoglycan interaction by non-anticoagulant heparin derivatives might interfere with the lymphatic outflow of leukocytes from the renal graft and might decrease the anti-graft allo-immune response.

## Methods

### Rat kidney transplantation model (Experiment 1)

Kidney allo-transplantation was performed from female Dark-Agouti (DA) donors to male DA or Wistar Furth (WF) recipients according to standard procedures as described previously [25]. The following experimental groups were included: control DA kidneys (N = 5), DA-to-DA isografts (N = 5), and DA-to-WF allografts (N = 5). Isograft recipients were sacrificed at day 81 (range 70–84) and allograft recipients at day 65 (range 54–84 days) after transplantation. The local animal ethics committee of the University of Groningen approved all the procedures used in the study and the Principles of Laboratory Animal Care (National Institute of Health publication no. 86–23) were followed.

### Laser dissection microscopy

Tubulo-interstitial tissue was dissected using the Leica Microdissection microscope (Leica Microsystems, Houston, Texas). RNA isolation and quantitative RT-PCR were performed essentially according to Asgeirsdottir *et al* [26]. Experimental details are described elsewhere [27]. Gene expression was analyzed with a custom made microfluidic card-based low density array (Applied Biosystems, Nieuwerkerk a/d IJssel, the Netherlands) using the ABI Prism 7900HT Sequence Detection System (Applied Biosystems, Nieuwerkerk a/d IJssel, the Netherlands). Composition of the low density array is indicated in Table 1. mRNA expression levels of CCL21 and 19 were measured separately in a qRT-PCR analysis. Final reaction volume was 10 μl consisting of 3μl cDNA, 5μl SYBR green Supermix (Bio-Rad, Veenendaal, The Netherlands), 0,8μl primer mix and 1,2 μl nuclease-free water. Reactions were performed using the ViiA^TM^ 7 Real-Time PCR System (Applied Biosystems, The Netherlands) at 95°C for 15s, 60°C for 15s, and 72°C for 5s, for 40 cycles. Relative mRNA levels were calculated as 2^−ΔCT^, in which ΔCT is CT_gene of interest_−CT_β-actin_.

**Table 1:** mRNA transcripts quantified in low density and regular qRT-PCR.

Gene name	General name	Gene symbol	Assay ID
**Lymphatic markers**			
Podoplanin	Podoplanin	*Pdpn*	Rn00571195_m1
**Proteoglycans**			
Perlecan	Perlecan	*LOC313641*	Rn01515780_g1
**Chemoattractants**			
Monocyte chemoattractant protein	CCL2	*Ccl2*	Rn00580555_m1
RANTES	CCL5	*Ccl5*	Rn00579590_m1
CX3CL1	CX3CL1	*Cx3cl1*	Rn00593186_m1
CXCL12	CXCL12	*Cxcl12*	Rn00573260_m1
CCL19	CCL19	*Ccl19*	Rn_Ccl19_1_SG
CCL21	CCL21	*Ccl21*	Rn_Ccl21_1_SG
**Reference Gene**			
Β-actin (2x)	Beta-actin	*Actb*	Rn00667869_m1

### Immunohistochemistry

Four μm acetone fixed frozen kidney sections were blocked for endogenous peroxidase activity with 0,03% H_2_O_2_. Sections were incubated for 1 hr with mouse anti-rat perlecan (10B2, kindly provided by Dr. Couchman, Biomedicine Institute, University of Copenhagen, Denmark) and rabbit anti-LYVE-1 (Millipore, Amsterdam, The Netherlands). For stainings LYVE-1 was chosen over podoplanin as lymph vessel marker due to cross-reactivity issues. Binding of primary antibody was detected by incubating the sections for 30 minutes with rabbit anti-mouse HRP (DAKO, Belgium) and goat anti-rabbit FITC (SouthernBiotech, USA) diluted in PBS containing normal rat serum. HRP activity was visualized using the Tetramethylrhodamine System (PerkinElmer LAS Inc). Nuclei were stained with DAPI. After the staining procedure photomicrographs were taken at 640x magnification.

### Heparin/heparin derivatives intervention study (Experiment 2)

In this study 38 female DA rats (donors) and 38 male WF rats (recipients) were used. Intervention was done with regular, unfractionated heparin (heparin, m.w. 21,116) and two non-anticoagulant heparin derivatives derived from heparin: N-desulfated and N-reacetylated heparin (N-acetylated heparin, m.w. 18,269) and periodate-oxidized, borohydride-reduced heparin (glycol split-heparin, m.w. 16,522). Production and characterization of these non-anticoagulant heparin derivatives have been described before [28,29]. The control transplanted group received daily vehicle (saline) injections. One day before transplantation, treatment with the respective formulations was started. The above mentioned groups daily received heparin/ non-anticoagulant heparin derivative between 9.00am and 12.00am dissolved in saline, injected subcutaneously at 2 mg/kg body weigh/day until sacrifice. The treatment dose was chosen according to previous studies [20,21] and is in the physiological range normally used for the treatment of thrombotic complications. Total follow up was 65±4 days (mean±SD). Animals were monitored on a daily basis for weight loss, activity and fur condition. When deteriorating condition of the animal was suspected, i.e. weight loss of >15% within 1 week and/or inactivity and/or worsened fur condition, a veterinarian was consulted and if necessary animals were sacrificed and regarded as drop outs. Upon sacrification animals were put under anesthesia and the vascular system was flushed with saline. During the follow up of 2–9 weeks, preterm graft loss occurred in 3 rats in the vehicle-treated group (N = 10), 5 in the unfractionated heparin group (N = 9), 2 in the glycol-split heparin group (N = 9) and 5 in the N-acetylated heparin group (N = 10). In total 15 animals dropped out of the experiment, of which 13 were sacrificed and 2 died without prior notice of illness. Graft loss was not significantly different among the groups.

### Immunohistochemistry

Four μm frozen and formalin-fixed paraffin embedded kidney sections were used for immunohistochemical stainings. Details on fixation, antigen retrieval, antibodies and conjugates are given in Table 2. All controls (omitting primary and/or secondary antibodies in various combinations) proved to be negative (not shown). As a lymphatic endothelium marker VEGFR3, podoplanin and LYVE-1 were tested. VEGFR3 appeared to be the most specific, i.e. no vessels were VEGFR3^+^ without being LYVE-1 or podoplanin positive. Hence, where possible, VEGFR3 was used as a lymphatic endothelial marker. When staining results were significantly better or double staining interactions prevented the use of VEGFR3, podoplanin or LYVE-1 was used as a lymphatic endothelial marker. After the staining procedure 20 photomicrographs were taken at 200x magnification from blood vessels or lymph vessels in cortical regions. Quantification of CD45^+^ leukocytes adherent to blood vessel endothelium, and leukocytes, MHCII^+^, ED-1^+^, CD3^+^, IDO^+^, HIS14^+^, CD103^+^ and HIS48^+^ cells inside lymphatic vessels was performed by using Aperio Imagescope software (Leica biosystems, Houston, Texas). The blood vessel and lymph vessel endothelium was encircled and endothelial circumference and area of the vessel were calculated. Counted cells were expressed as number of cells per 100 μm of endothelium. Interstitial inflammation was determined by measuring the amount of CD45^+^ nucleated cells in the renal cortex using the TissueFAXS software (Tissuegnostics, Vienna, Austria). Data is expressed as number of nucleated cells positive for CD45 in the renal cortex. Lymphangiogenesis was determined by staining transplanted and non-transplanted kidneys of vehicle treated animals and counting the amount of lymph vessels per high power field. 10 photomicrographs per animals were randomly taken at 100x magnification and manually quantified. CCL21 expression in the kidney was determined using 30 photomicrographs per animal which were randomly taken at 100X magnification. Quantification was done using the MacBiophotonics ImageJ program (Rasband, W.S., ImageJ, U.S. National Institute of Health, Bethesda, Maryland, USA). Data are expressed as % positive stained surface area.

**Table 2:** Immunohistochemical staining characteristics.

Cell type	Tissue processing	Marker	Antibody	Conjugate + Visualization
**Lymphatic endothelium**	Formaldehyde fixed, paraffin embedded, deparaffinization, Tris/EDTA (pH 9.0)	Podoplanin	Mouse anti-rat Podoplanin, (Angiobio, Huissen, Netherlands) 1:100	Goat anti mouse Ig PO (Southern Biotech, Birmingham, USA); 1:100, 3,3’-Diaminobenzidine (DAB) (DAKO, Glostrup, Denmark) followed by PAS staining
**Lymphatic endothelium**	Cryosections, Acetone fixed	VEGFR3	Goat anti-mouse VEGFR3 (R&D systems, Minneapolis, USA) 1:40	Rabbit anti- goat Ig HRP (DAKO, Glostrup, Denmark) 1:100. Tetramethylrhodamine System (PerkinElmer LAS Inc)
**Vascular endothelium + Leukocytes**	Cryosections, Acetone fixed	Van Willebrand factor + CD45	Rabbit anti-human vWF (Abcam, Cambridge, UK) 1:400 + Mouse anti-rat CD45 antibody (clone OX-1) 1:4	Goat anti-Rabbit Ig-FITC (DAKO, Glostrup, Denmark) 1:50 + Goat anti-mouse Ig HRP 1:100 (DAKO, Glostrup, Denmark). Tetramethylrhodamine System (PerkinElmer LAS Inc)
**Lymphatic endothelium + APC’s**	Cryosections, Acetone fixed	VEGFR3 + MHCII	Goat anti-mouse VEGFR3 (R&D systems, Minneapolis, USA) 1:40 + Mouse anti-rat MHCII (Hycult biotech) 1:100	Rabbit anti-mouse Ig Biotin (DAKO, Glostrup, Denmark) 1:100 + Rabbit anti- goat Ig HRP 1:100 (DAKO, Glostrup, Denmark). Strepavidin-FITC 1:25 (DAKO, Glostrup, Denmark) + Tetramethylrhodamine System (PerkinElmer LAS Inc)
**Lymphatic endothelium + Macrophages**	Cryosections, Acetone fixed	VEGFR3 + ED-1	Goat anti-mouse VEGFR3 (R&D systems, Minneapolis, USA) 1:40 + mouse anti-rat CD68 antibody (clone ED-1), (abd serotech, Oxford, UK), 1:500.	Rabbit anti-mouse Ig Biotin (DAKO, Glostrup, Denmark) 1:100 + Rabbit anti- goat Ig HRP 1:100 (DAKO, Glostrup, Denmark). Strepavidin-FITC 1:25 (DAKO, Glostrup, Denmark) + Tetramethylrhodamine System (PerkinElmer LAS Inc)
**Lymphatic endothelium + T-cells**	Cryosections, Acetone fixed	VEGFR3 + CD3	Goat anti-mouse VEGFR3 (R&D systems, Minneapolis, USA) 1:40 + Rabbit anti-human CD3; (DAKO, Glostrup, Denmark) 1:100.	Mouse anti-rabbit IgG HRP (DAKO, Glostrup, Denmark) 1:100 + Rabbit anti- goat Ig FITC 1:50. Tetramethylrhodamine System (PerkinElmer LAS Inc)
**Lymphatic endothelium + Neutrophils**	Cryosections, Acetone fixed	VEGFR3 + HIS48	Goat anti-mouse VEGFR3 (R&D systems, Minneapolis, USA) 1:40 + Mouse anti-rat HIS48 (Kindly provided by prof. dr. Hillebrands) 1:2.	Rabbit anti-mouse IgM HRP (Southern Biotech, Birmingham, USA) 1:100 + Rabbit anti- goat Ig FITC 1:50. Tetramethylrhodamine System (PerkinElmer LAS Inc)
**Lymphatic endothelium + Indoleamine 2, 3-dioxy-genase (IDO)**	Cryosections, Acetone fixed	VEGFR3 + IDO	Goat anti-mouse VEGFR3 (R&D systems, Minneapolis, USA) 1:40 + Rabbit anti-Human IDO (Millipore, Amsterdam, The Netherlands) 1:50	Mouse anti-rabbit IgG HRP (DAKO, Glostrup, Denmark) 1:100 + Donkey anti-Goat IgG (Southern Biotech, Birmingham, USA) 1:50. Tetramethylrhodamine System (PerkinElmer LAS Inc)
**Lymphatic endothelium + B-cells**	Cryosections, Acetone fixed	VEGRFR3 + HIS14	Goat anti-mouse VEGFR3 (R&D systems, Minneapolis, USA) 1:40 + Mouse anti-Rat HIS14 (Kindly provided by prof. dr. Hillebrands) 1:1000. [30]	Donkey anti-Goat IgG (Southern Biotech, Birmingham, USA) 1:50. + Goat anti-Mouse IgG1 HRP (Southern Biotech, Birmingham, USA) 1:100. Tetramethylrhodamine System (PerkinElmer LAS Inc)
**Lymphatic endothelium + Dendritic-cells**	Cryosections, Acetone fixed	Lyve-1 + OX-62	Sheep anti-rat Lyve-1 (R&D systems, Minneapolis, USA) 1:100 + Mouse anti-rat CD103 (OX-62)(Hercules, CA, USA) 1:100	Rabbit anti-sheep FITC (Abcam, Cambridge, UK) 1:50 + Goat anti-mouse IgG1 HRP (Southern Biotech, Birmingham, USA) 1:100. Tetramethylrhodamine System (PerkinElmer LAS Inc)
**CCL21 expression**	Cryosections, Acetone fixed	CCL21	Goat anti-mouse CCL21 (R&D systems, Minneapolis, USA) 1:20	Rabbit anti-Goat HRP (DAKO, Glostrup, Denmark) 1:100, Tetramethylrhodamine System (PerkinElmer LAS Inc)

Quantification of interstitial fibrosis was done by staining sections in the following manner. Four μm formalin fixed and paraffin embedded sections were deparaffinized and incubated in Picro Sirius red solution (0,1g Sirius red in 100 ml picric acid) for 1 hour. Thereafter sections were incubated in 0,01 N HCl for 2 minutes, dehydrated and covered using DEPEX (Klinipath BV, Duiven, The Netherlands) mounting medium. Per kidney 25 photomicrographs were randomly taken at 200x magnification and digitally analyzed using MacBiophotonics ImageJ program (Rasband, W.S., ImageJ, U.S. National Institute of Health, Bethesda, Maryland, USA). The amount of interstitial fibrosis was expressed as % positive surface area.

### ELISA competition assay

We used ELISA to evaluate whether heparin/ non-anticoagulant heparin derivatives compete with the binding of CCL2 and CCL21 to perlecan. For that purpose Maxisorp 96-well flat bottom microtiter plates (U96 from VWR International, Amsterdam, The Netherlands) were coated overnight in PBS with 5 μg perlecan/ml PBS (Sigma, Zwijndrecht, The Netherlands). After washing in PBS, wells were blocked with 5% skimmed milk powder in PBS for 1 h. In a separate microtiter plate, 1,25 μg/ml human recombinant CCL2 or 0,7 μg/ml recombinant CCL21 (both: PeproTech, Hamburg, Germany) were incubated with a dilution range of either unfractionated heparin, N-acetylated heparin or glycol-split-heparin for 30 min, then transferred to the ELISA plate and incubated for 1 h. The concentrations of CCL2 (1.25 μg/ml) an CCL21 (0,7 μg/ml) were chosen in order to achieve an ELISA signal of around 1.5 OD. The wells were washed again, and respectively monoclonal mouse anti-human CCL2 IgG1 (1:1000; eBioscience, Frankfurt, Germany) or Goat anti- mouse CCL21 IgG (1:500, R&D systems, Minneapolis, USA) diluted in 1% skimmed milk powder was added for 1 h. After washing, HRP-labeled goat anti-mouse IgG1, 1:500 (Southern biotech, Birmingham, USA) or Rabbit anti- Goat Ig, 1:500 (DAKO, Glostrup, Denmark) was added. Substrate reaction was done with 3,3’,5,5’-tetramethylbenzidine substrate (Sigma, Zwijndrecht, The Netherlands) for 15 min in the dark, and the reaction was stopped by adding 1.5 N H2SO4. Absorbance was measured at 450 nm in a microplate reader. All incubations were done at room temperature in a volume of 100μl/well. All experiments were performed three times in duplicate.

### Statistics

mRNA expression levels were analyzed using a one-way ANOVA with Tukey’s post hoc test. In the rat transplantation experiment, differences between the groups were analyzed using a Mann Whitney U test adjusted for multiple comparisons. P<0.05 was considered statistically significant. The inhibitory effect of heparinoids in vitro and lymphangiogenesis were analyzed by a two-way ANOVA. Spearman correlation analysis was performed to show correlations between histological parameters and kidney function.

## Results

### Experiment 1

To show lymphangiogenesis, identify the presence of perlecan in the lymphatic basement membrane and measure the lymphatic chemokine expression profile in renal transplantation we counted VEGFR3 positive lymph vessels in non-transplanted donor and allografted kidneys and evaluated untreated allograft, isograft and control kidneys for perlecan, podoplanin and chemokine expression.

#### Perlecan is a constitute of lymphatic basement membrane and is upregulated in CTD-associated lymphangiogenesis

**Fig 1:**
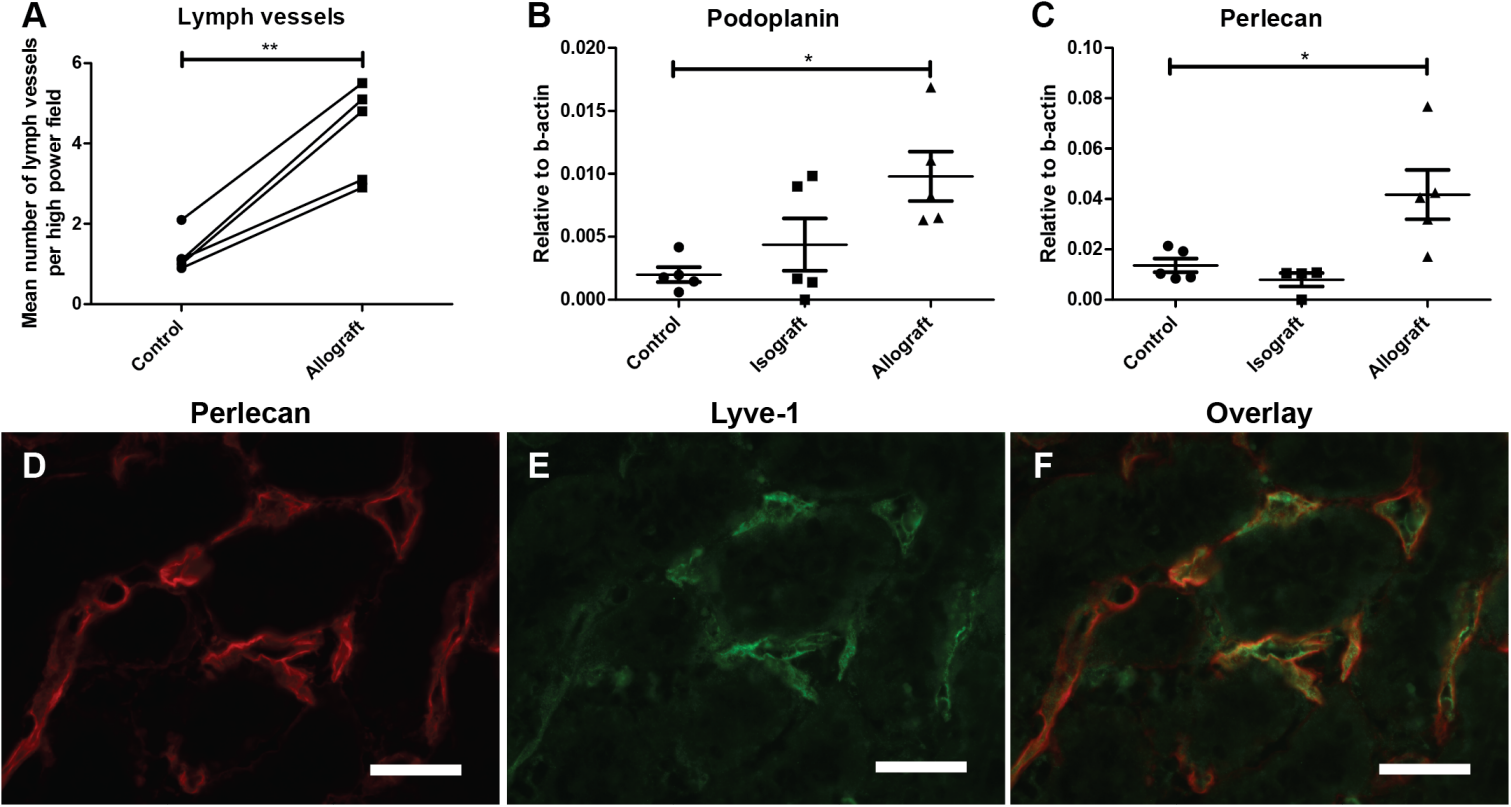
CTD in allografted kidneys is accompanied by lymph vessel formation. (A) Counting of VEGFR3+ lymph vessels revealed a threefold increase in allografted kidneys compared to the non-transplanted contralateral kidneys from the same donor rats (p<0.01). qRT-PCR of podoplanin and perlecan in dissected tubulo-interstitium revealed an increase in the expression of the (B) lymphatic endothelial marker podoplanin and (C) the vascular basement membrane marker perlecan in allografted kidneys (N = 5) compared to non-transplanted control kidneys (N = 5)(both p<0,05). Data are presented as mean ± SEM. DA-to-DA isografted kidneys (N = 5) did not show an increase in the expression of podoplanin and perlecan. Double staining for perlecan (D) and lymphatic marker LYVE-1 (E) revealed colocalization of perlecan and lymphatic endothelium (F).

Counting the number of VEGFR3+ lymph vessels in non-transplanted control kidneys and untreated allografted kidneys shows a marked increase in lymph vessels in allografted kidneys (Fig 1A). Double staining for perlecan and lymph vessels by LYVE-1 showed perlecan in vascular basement membranes. Partial colocalization of perlecan with the LYVE-1 positive lymphatic endothelium reveals perlecan being a constituent of the lymphatic basement membrane (Fig 1D–1F). qRT-PCR of laser dissected tubulo-interstitium of allografted kidneys showed an increase in both perlecan and podoplanin expression compared to non-transplanted controls (both p<0,05)(Fig 1B and 1C). The increase of VEGFR3+ lymph vessels and an increased expression of both perlecan and podoplanin shows that in transplantation there is an ongoing expansion of the lymphatic system.

#### Tubulo-interstitial chemokine expression is increased in renal transplantation

**Fig 2:**
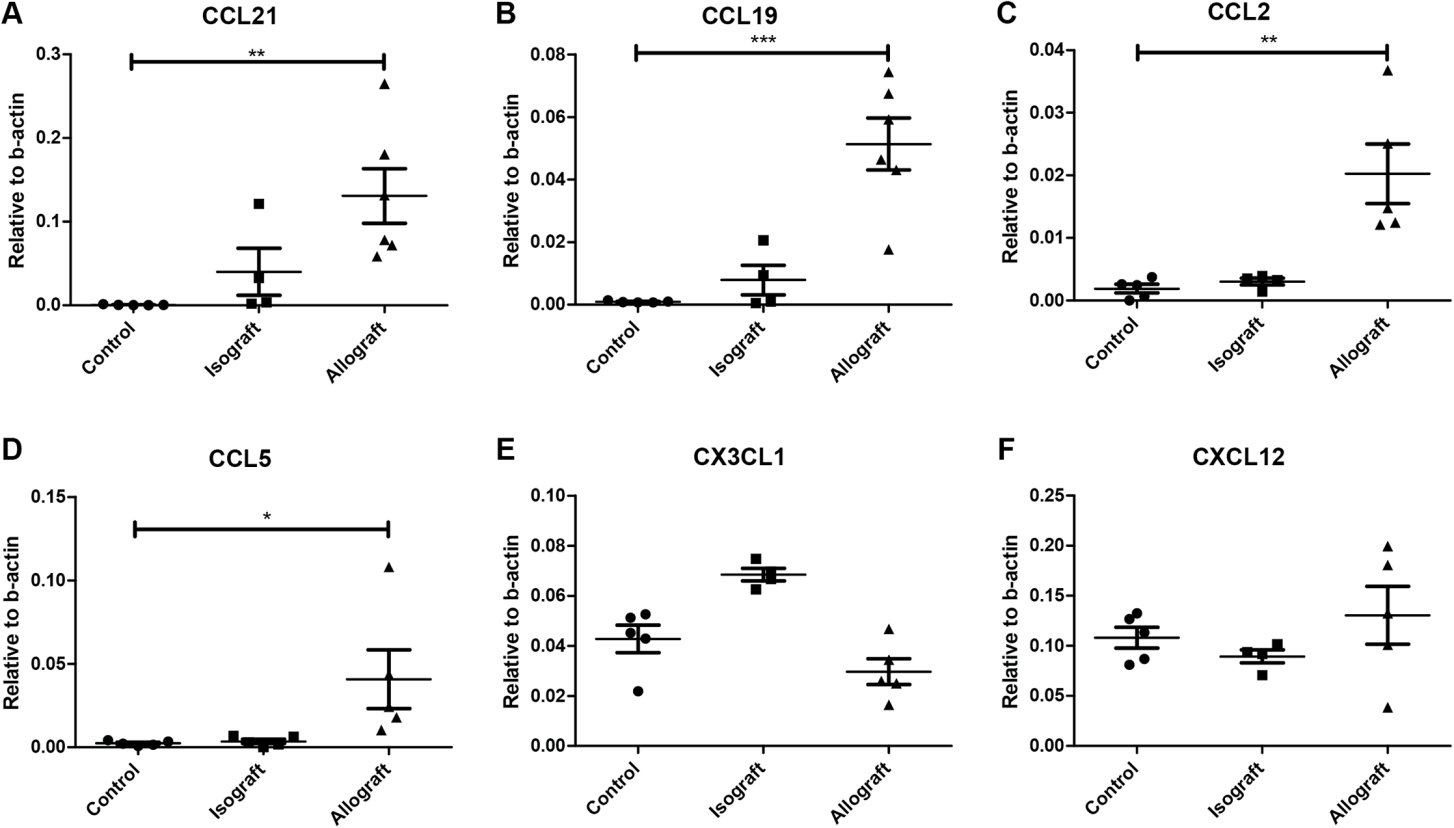
Tubulo-interstitial upregulation of lymphatic chemokines. qRT-PCR for lymphatic chemokines in laser dissected tubulo-interstitium reveals a strong increase of CCL21, CCL19, CCL2 and CCL5 in allografted kidneys (N = 5) compared to healthy control kidneys (N = 5) (P<0,01, P<0,001,P<0,01 and P<0,05 respectively). No upregulation was seen in CX3CL1 and CXCL12 expression in allografts. Isografted kidneys (N = 5) showed upregulation of only CX3CL1. Experiments were done in triplicate. Data are presented as mean ± SEM.

qRT-PCR of laser dissected tubulo-interstitium was performed to assess whether newly-formed lymph vessels were active in producing chemokines. Allograft kidneys show a markedly increased expression of CCL21 and CCL19 compared to non-transplanted controls (p<0.01, p<0.001 resp.)(Fig 2A and 2B). Since CCL21 is considered the most important chemokine for lymphatic migration, these results suggest functionality of the newly formed lymph vessels. CCL2 and CCL5 are most known for their role in leukocyte influx from the vascular system into the interstitium. It is however known that they also play a role in leukocyte efflux towards lymph vessels. In allograft kidneys both CCL2 and CCL5 expression is upregulated in the tubulo-interstitium compared to non-transplanted controls (p<0.01, p<0.05 resp.)(Fig 2C and 2D). It is however not known whether the rise in CCL2 and CCL5 expression can be accounted to either vascular or lymphatic endothelial cells or both. Chemokines CX3CL1 and CXCL12, which can also be involved in lymphatic leukocyte migration, do not show increased expression in allografted kidneys (Fig 2E and 2F). These results show that lymphatic endothelium specific chemokines are upregulated in the tubulo-interstitium of renal allografts.

#### Newly formed lymph vessels in renal transplantation contain predominantly APCs

**Fig 3:**
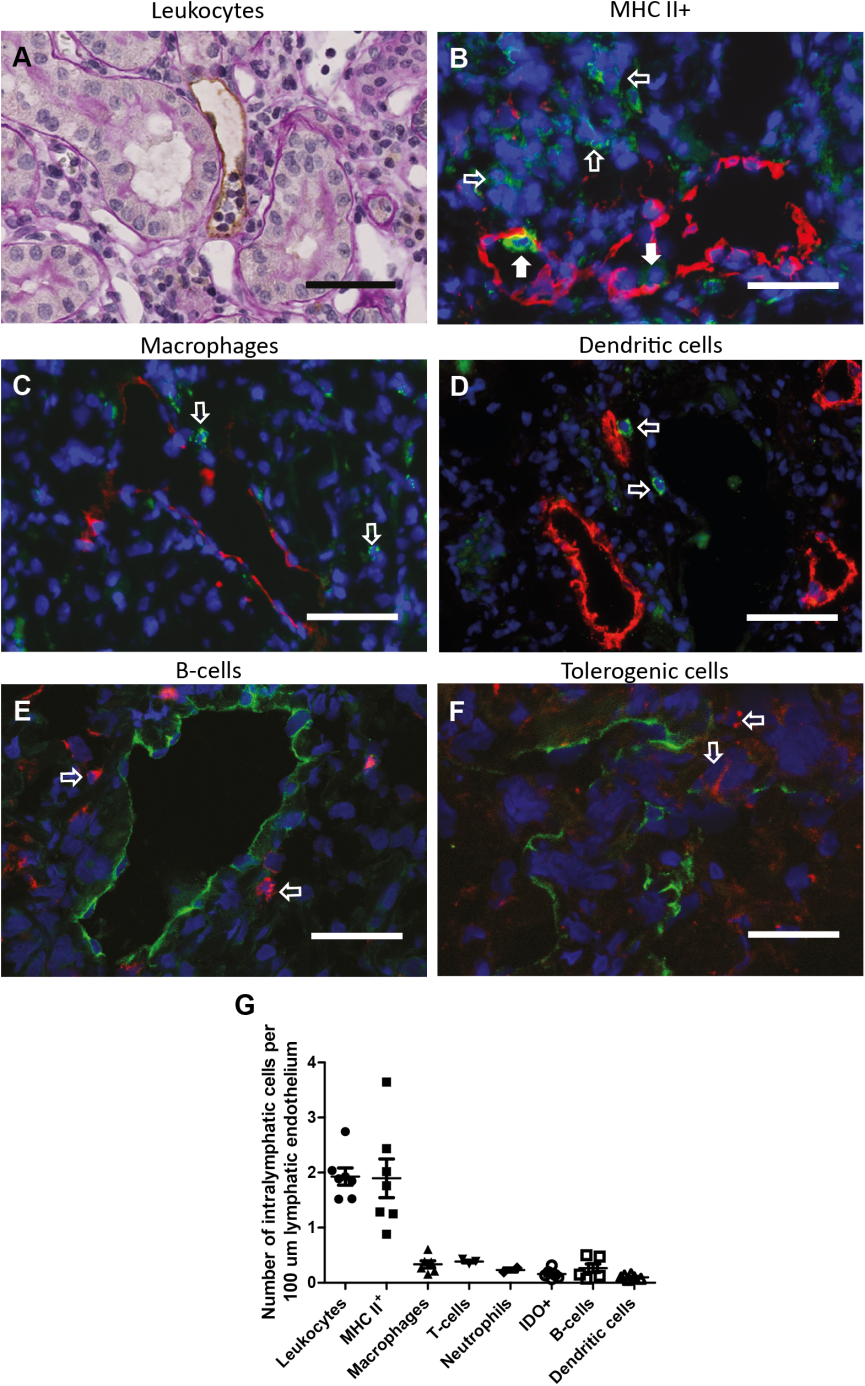
Intra-lymphatic leukocytes in CTD are mainly HLA-II^+^, ED-1^-^, IDO^-^, CD103^-^ and HIS14^-^ APCs. Intra-lymphatic leukocytes were scored by counting intra luminal nucleated cells in a podoplanin/PAS double staining (A). Intra lymphatic MHCII^+^ cells (B)(green), macrophages (ED-1)(C)(green),dendritic cells (CD103)(D)(green), B-cells (HIS14)(E)(red) and tolerogenic cells (IDO)(F)(red) were scored in a double staining with the lymphatic marker VEGFR3 or Lyve-1. Closed arrows point out intralymphatic accumulation of cells positive for the respective staining. Open arrows indicate presence of cells positive for the respective staining outside the lymphatic system. Comparing the intra-lymphatic amount of different leukocyte species in untreated renal allografts (N = 7) revealed that the main cell accumulating in the tubulo-interstitium is MHCII^+^, ED-1^-^, IDO^-^, CD103^-^ and HIS14^-^ (G), likely representing antigen presenting cells. Scale bars represent 50μm. Data are presented as mean ± SEM.

Leukocyte accumulation in lymph vessels was scored to determine whether newly formed lymph vessels are functional. Scoring showed an increase in leukocyte accumulation in lymph vessel lumen. Intra luminal nucleated cells were seen in approximately 90% of the lymph vessels scored, giving a strong indication that newly formed lymph vessels were active in accumulating leukocytes (Fig 3A). To investigate which leukocyte subtypes are exiting the interstitium via the lymphatic route we scored MHCII^+^ cells (Fig 3B), macrophages (ED-1)(Fig 3C), dendritic cells (CD103)(Fig 3D), B-cells (HIS14) (Fig 3E), tolerogenic cells (IDO) (Fig 3F), T-cells (CD3) and neutrophils (HIS48) in the lumen of cortical lymphatic vessels. The results showed intra-lymphatic cells to be mainly MHCII^+^, ED-1^-^, CD103^-^ and HIS14^-^ suggesting antigen-presenting cells are predominantly accumulating in lymph vessels in CTD (Fig 3G). Like ED-1^+^ macrophages, IDO^+^ tolerogenic cells and HIS14^+^ B-cells, T-cells and neutrophils are scarce in the lymphatic lumen.

### Experiment 2

To evaluate the effect of heparin/ non-anticoagulant heparin derivatives on inflammation, lymphatic migration and kidney function, transplanted animals were treated daily with the heparin/ non-anticoagulant heparin derivatives. Inflammation, lymphatic leukocyte accumulation and renal function were determined.

#### Treatment of renal transplanted rats with glycol split heparin reduced renal leukocyte recruitment

**Fig 4:**
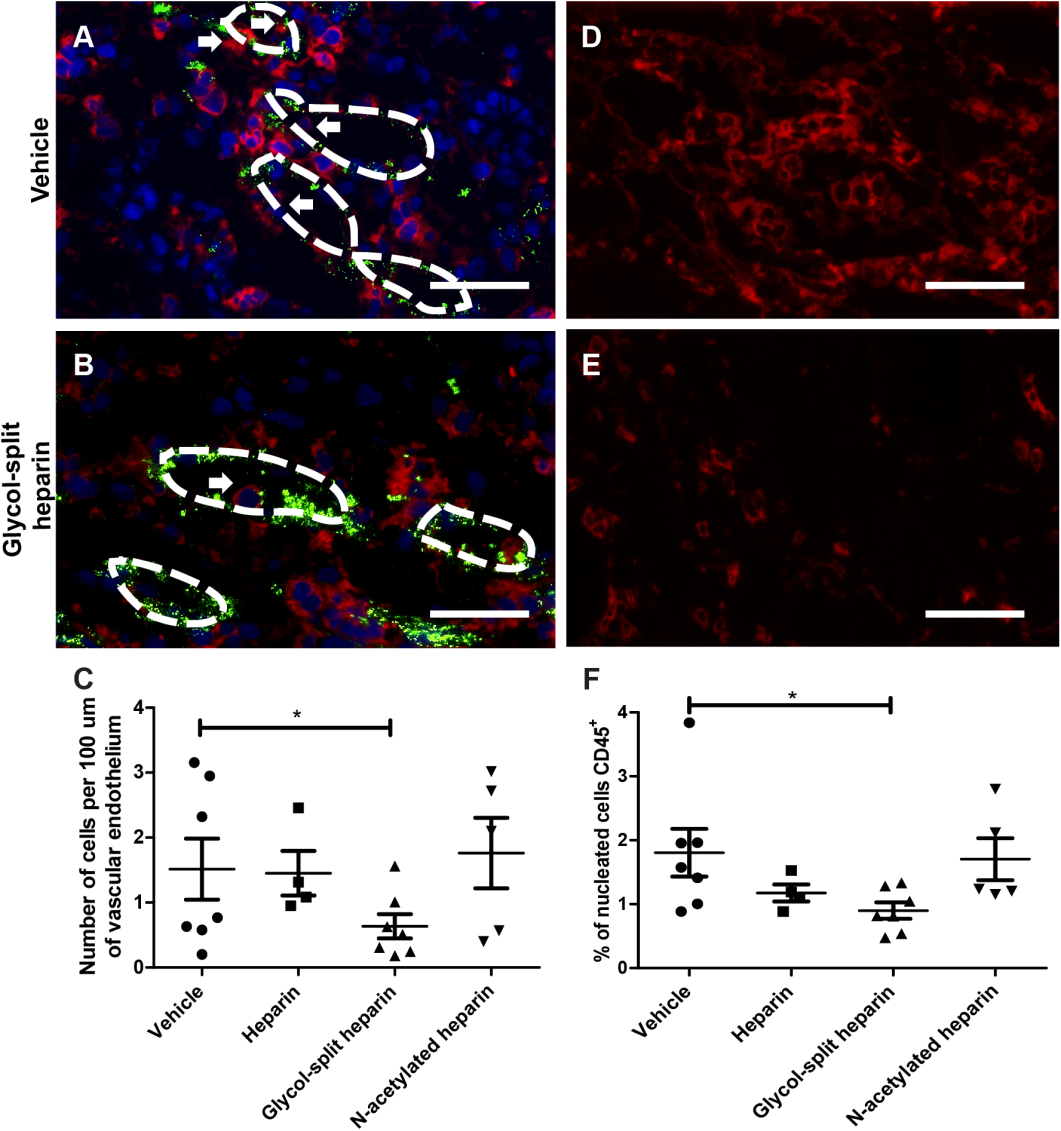
Effect of heparin/non-anticoagulant heparin derivative treatment on leukocyte influx in rat renal transplantation. Adhesion (white arrows) of leukocytes (CD45 in red) to blood vessel endothelium (vWF in green) in kidney allografts is reduced in the glycol-split heparin treated rats (N = 7) compared to the vehicle treated rats (N = 7) (p<0.05) (A-C). Moreover, glycol-split heparin treatment (N = 7) also reduces leukocyte count in the interstitium of transplanted rat kidneys (CD45 in red), compared to vehicle treated animals (N = 7) (p<0.05) (D-F). No significant differences were seen between heparin (N = 4) or N-acetylated heparin treated animals (N = 5) compared to vehicle treated animals (N = 7). Dotted lines represent vessel lumen. Scale bars represent 50μm. Data are presented as mean ± SEM.

Glycosaminoglycans have been shown to have anti-inflammatory potential. To investigate whether heparin/non-anticoagulant heparin derivative treatment could reduce renal leukocyte recruitment, we scored the number of CD45^+^ leukocytes adhered to vascular endothelium, visualized by a van Willebrand factor staining. Adjusted for vascular endothelial length, glycol-split heparin-treated animals showed a significant reduction in the number of CD45^+^ leukocytes adherent to the vascular endothelium compared to vehicle-treated animals (p<0.05)(Fig 4A–4C). No differences were seen between the vehicle treated group and either the unfractionated heparin or the N-acetylated heparin group. To assess whether the reduction in endothelial leukocyte adherence in the glycol-split heparin group resulted in a reduction in the tubulo-interstitial inflammation, CD45^+^ cells in the interstitium were scored. Values are expressed as the percentage of CD45^+^ cells in the renal cortex. In the tubulo-interstitium the glycol-split heparin group also showed a reduction in leukocyte influx compared to vehicle treatment (Fig 4D–4F). No differences were seen between the vehicle treated group and either the unfractionated heparin or the N-acetylated heparin group. Scoring of interstitial leukocyte subtypes was performed for T-cells (CD3), neutrophils (HIS48), MHC II^+^ cells, dendritic cells (CD103) and macrophages (ED-1), however no differences between treatment groups were found (data not shown).

#### Treatment of renal transplanted rats with glycol split heparin increased APC accumulation in lymph vessels and worsened renal outcome

To investigate whether glycol-split heparin treatment and the subsequent reduction of inflammation resulted in a better graft function we measured plasma creatinine and urea and determined creatinine clearance. Treatment with heparin/ non-anticoagulant heparin derivatives did not alter survival nor resulted in improved transplant function. Moreover, it seems to worsen kidney function in the glycol-split heparin treated group. Although not significant, creatinine clearance seems to be lower in glycol-split treated animals compared to vehicle treated animals (0,4 and 0,6 ml/min resp.) and plasma urea is higher (43 and 28 mmol/L resp.). Also kidney hypertrophy is the highest in the glycol-split heparin treated group (Table 3).

**Table 3:** Kidney function parameters measured at baseline, 4 and 8 weeks after transplantation. Plasma and urine was collected at baseline, 4 and 8 weeks after transplantation. Both plasma creatinine and urea rose during follow-up after transplantation while the creatinine clearance was reduced in all groups. Although not significant, glycol-split heparin treatment seemed to result in higher plasma creatinine and urea and lower creatinine clearance compared to vehicle treated animals. Also kidney hypertrophy, a measure for worsening kidney condition, was highest in glycol-split heparin treated animals.

	Timepoint	Vehicle (n = 7)	Heparin (n = 4)	Glycol-split heparin (n = 7)	Nac heparin (n = 5)
Δ kidney weight		1,3(0,4–2,3)	1,2(0,9–1,5)	1,5(0,4–3,1)	1,4(1–1,7)
Plasma creatinine (μmol/L)	Baseline	19(18–21)	19(19–21)	15(14–17)	15(15–19)
	4 weeks after Tx	73(59–120)	64(52–73)	65(54–168)	81(53–84)
	8 weeks after Tx	96(73–139)	72(58–99)	136(70–207)	73(58–122)
Creatinine Clearance (ml/min)	Baseline	3,0(2,8–3,8)	2,6(2,5–3,0)	3,5(2,7–3,7)	3,0(2,3–3,0)
	4 weeks after Tx	0,7(0,4–1,1)	1,0(1,0–1,3)	1,0(0,5–1,2)	0,7(0,1–0,8)
	8 weeks after Tx	0,6(0,3–1,0)	0,8(0,5–1,2)	0,4(0,2–0,9)	0,9(0,4–1,0)
Plasma urea (mmol/L)	Baseline	6(5–7)	6(5–7)	6(5–6)	6(5–6)
	4 weeks after Tx	20(18,3–36)	18(16–20)	20(14–39)	19(15–24)
	8 weeks after Tx	28(21–51)	22(18–37)	43(21–64)	27(24–40)

**Fig 5:**
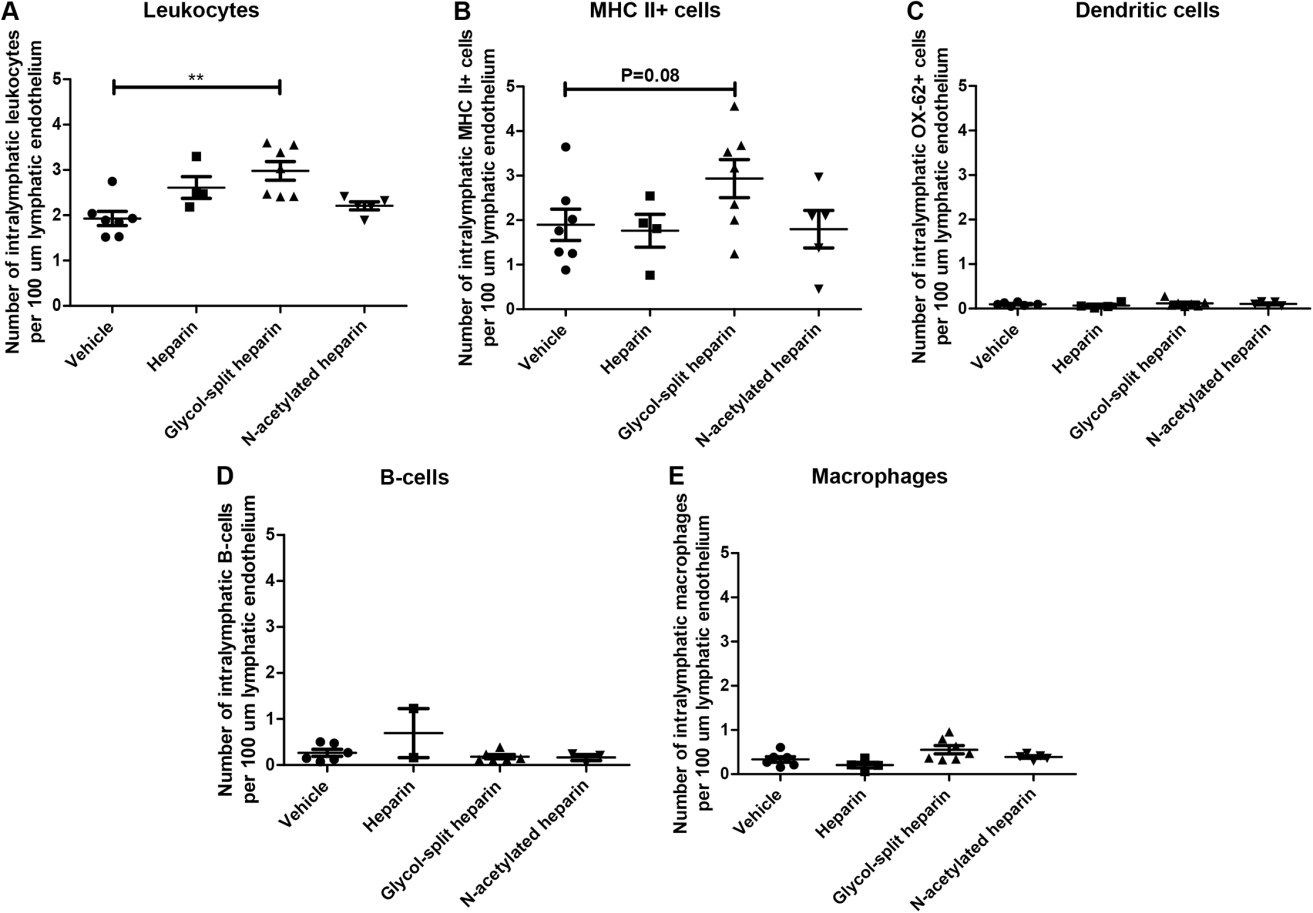
Glycol-split heparin treatment increased intra-lymphatic leukocyte accumulation. Nucleated cells on the luminal side of lymph vessels were identified and counted to assess the intralymphatic accumulation of leukocytes in heparin/non-anticoagulant heparin derivative treated allograft kidneys. Glycol-split heparin treatment resulted in an increased lymphatic leukocyte accumulation compared to vehicle treated animals (p<0,01)(A). Data is expressed as the number of cells per 100 μm of endothelium. Staining for both lymphatic endothelium and MHCII revealed a borderline increase of MHCII positive cells in the lymphatic lumen in glycol-split heparin treated (N = 7) compared to vehicle treated animals (N = 7) (p = 0,08) (B). Intralymphatic scoring of CD103^+^ dendritic cells, HIS14^+^ B-cells and ED-1^+^ macrophages did not reveal any effect of heparin/non-anticoagulant heparin derivative treatment on the intraluminal presence of these cells (C-E). No significant differences were seen between heparin (N = 4) or N-acetylated heparin treated animals (N = 5) compared to vehicle treated animals (N = 7) in either of the intralymphatic cell populations. Scoring was performed as in Fig 3. Data are presented as mean ± SEM.

To explain why a reduction in inflammation did not lead to a reduced alloresponse and subsequently to an improved graft function in the glycol-split heparin group, we investigated the effect of heparin/ non-anticoagulant heparin derivatives treatment on leukocyte efflux from the interstitium towards the lymphatic system. In a podoplanin/periodic acid—Schiff double staining the number of leukocytes on the luminal side of the lymphatic endothelium was scored. Significantly more leukocytes were accumulated on the luminal side of the lymphatic endothelium in the glycol-split heparin group compared to the vehicle treated group (p<0.01) (Fig 5A). Since we showed earlier that the major leukocyte subtype that accumulates in the lymphatics is MHCII^+^, we also quantified whether heparin/ non-anticoagulant heparin derivative treatment could decrease MHCII+ cell accumulation in the lymphatic lumen. The results showed that accumulation of MHCII^+^ APCs is borderline increased in glycol-split heparin treated group compared to vehicle treated animals (p = 0,08) (Fig 5B). Heparin and N-acetylated heparin treated did not show an effect on intra-lymphatic MHCII^+^ cell accumulation. Staining for dendritic cells (CD103), macrophages (ED-1) and B-cells (HIS14) revealed a lower mean number of dendritic cells, macrophages and B-cells in the lymphatic lumen compared to MHC II+ APCs in all treatment groups and vehicle treated animals. Moreover, no effect of treatment was seen in dendritic cell, macrophage and B-cell accumulation in the lymphatic lumen (Fig 5C–5E). Also neutrophil (HIS48), tolerogenic cells (IDO) and T-cell (CD3) accumulation in the lymphatic lumen did not show treatment induced alterations (data not shown).

#### Glycol split heparin inhibited binding of CCL21 to immobilized perlecan in vitro and dispersed CCL21 in vivo

**Fig 6:**
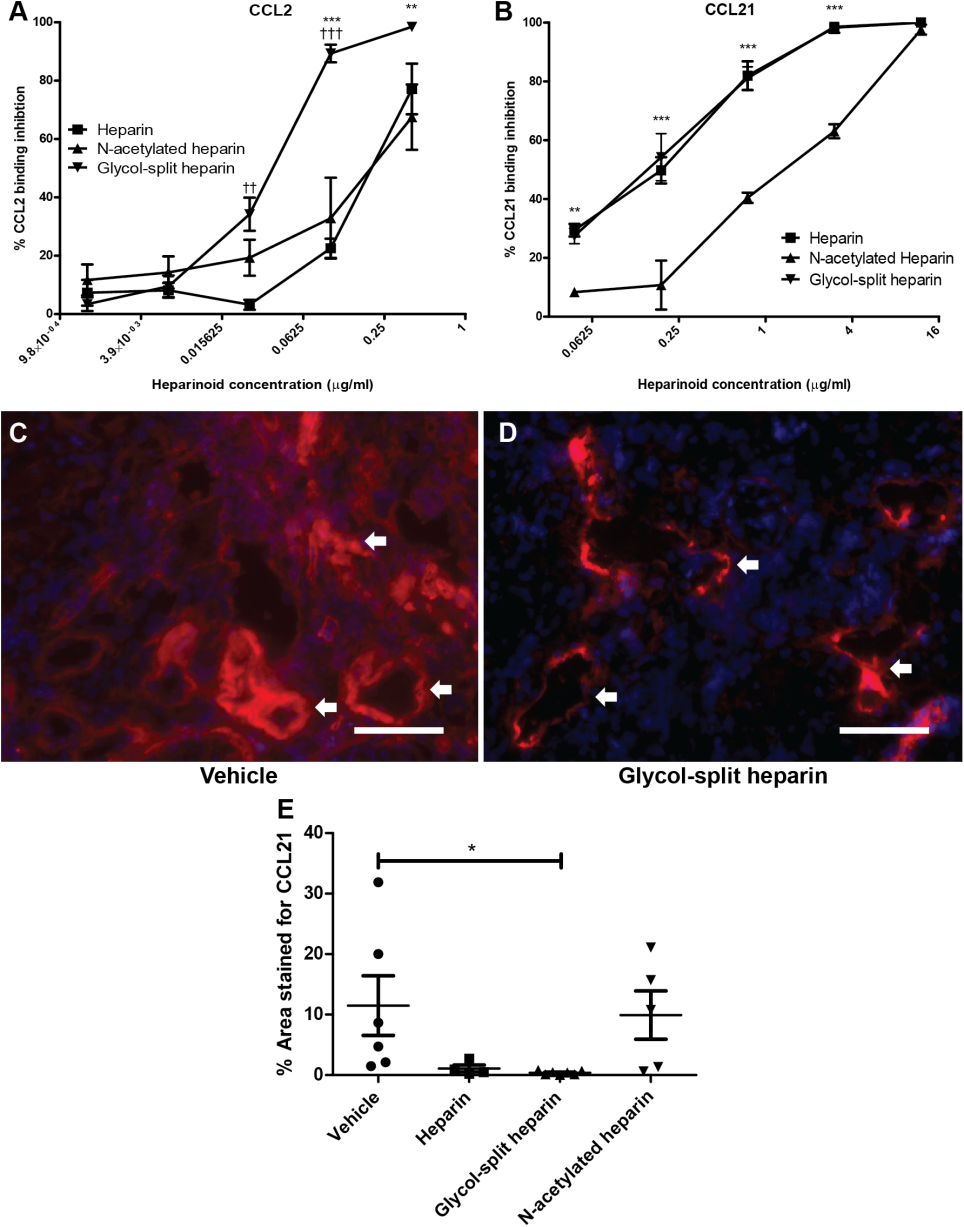
Glycol-split heparin revealed a high binding affinity for chemokines resulting in reduced (peri-)lymphatic CCL21 presence in glycol-split heparin treated kidneys. To determine whether heparin/non-anticoagulant heparin derivatives are capable of competing with the CCL2 and CCL21 interaction with perlecan, CCL2 or CCL21 were incubated together with a concentration range of heparin/non-anticoagulant heparin derivatives on a perlecan coated plate. Residual CCL2 and CCL21 binding to the immobilized perlecan was measured and revealed that glycol-split heparin has the highest affinity for CCL2 and CCL21 (A+B). Significant differences are expressed as glycol-split heparin compared to heparin (†) and N-acetylated heparin (*). Experiments were done 3 times in duplicate. *In vivo* treatment with glycol-split heparin (N = 7) resulted in a reduced CCL21 staining of (peri-)lymphatic areas compared to vehicle treated animals (N = 7) (C-E). Lymph vessels are pointed with white arrows. Scale bars represent 50μm. * = p<0,05, ††/** = p<0,01, †††/*** = p<0,001. Data are presented as mean ± SEM.

To explain why treatment with glycol-split heparin both leads to reduced leukocyte influx but increased accumulation in lymph vessels we tested in vitro if heparin/ non-anticoagulant heparin derivative treatment could inhibit the binding of CCL2 and CCL21 to immobilized perlecan. In this experiment we co-incubated CCL2 or CCL21 with increasing amounts of the heparin/ non-anticoagulant heparin derivatives and measured the binding of these chemokines to immobilized perlecan. Glycol-split heparin shows the strongest inhibition of CCL2 binding to perlecan whereas heparin and N-acetylated heparin show equal ability to inhibit CCL2 binding to perlecan (Fig 6A). Glycol-split heparin and heparin show equal ability to inhibit the binding of CCL21 to perlecan. Of the non-anticoagulant heparin derivatives, N-acetylated heparin shows the lowest affinity for CCL21 (Fig 6B), which can be explained by the lower sulfation degree of N-acetylated heparin compared to heparin and glycol-split heparin.

To test whether treatment with heparin/ non-anticoagulant heparin derivatives had an effect on the tissue distribution of CCL21, CCL21 was stained and quantified in the renal cortex. In the unmodified heparin and glycol-split heparin group CCL21 positivity was predominantly seen in vessel like structures, likely lymph vessels (Fig 6D). In contrast, vehicle and N-acetyl heparin treated animals showed a more expanded staining in the peri-lymphatic interstitium (Fig 6C), besides a lymphatic endothelial staining pattern like in heparin and glycol-split heparin. Quantification of CCL21 in the renal cortex revealed a reduced CCL21 expression in the glycol-split heparin group compared to vehicle treated animals (Fig 6E). This might imply that the strong affinity of heparin and glycol-split heparin for CCL21 causes CCL21 to disperse from the lymphatic endothelium and its direct periphery, expanding the chemotactic gradient.

#### Intra-lymphatic leukocytes, rather than interstitial leukocytes or fibrosis correlated with reduced allograft function

**Fig 7:**
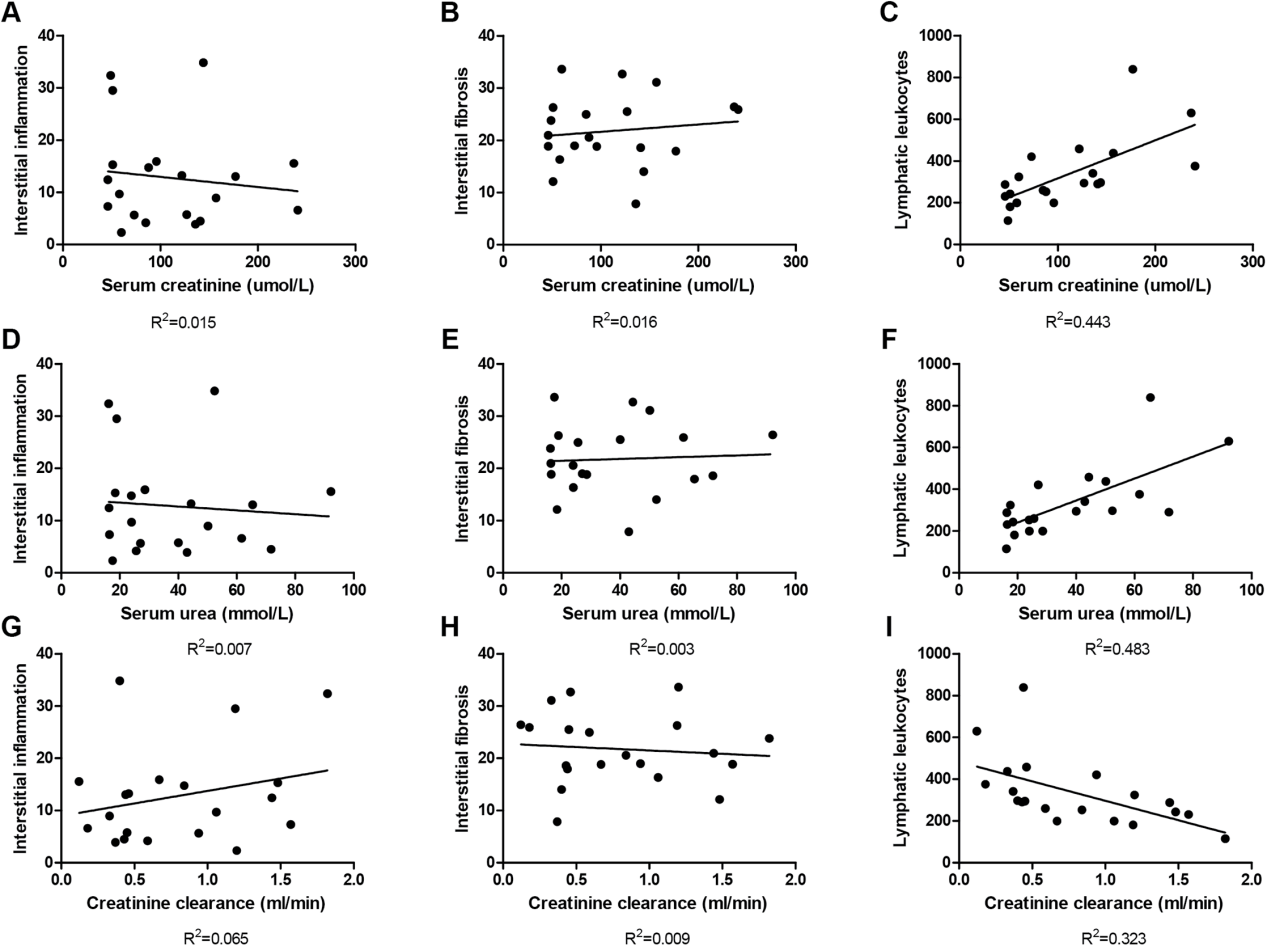
Kidney function correlated with lymphatic leukocytes. Correlation analysis between kidney function (plasma creatinine, plasma urea and creatinine clearance) and histological parameters (interstitial inflammation, interstitial fibrosis and lymphatic leukocytes) revealed that there is no correlation between kidney function and interstitial inflammation or interstitial fibrosis. However, there was a strong correlation between kidney function and the total number of intra lymphatic leukocytes (C: p = 0.001, F: p<0.001, I: p<0.01).

To evaluate potential implications of leukocyte efflux towards lymph vessels we performed correlation analyses. We correlated serum creatinine, serum urea and creatinine clearance as renal function parameters with interstitial leukocyte numbers, interstitial fibrosis and total intra-lymphatic leukocyte numbers. Serum creatinine levels failed to show a correlation with interstitial inflammation and interstitial fibrosis (r^2^ = -0.278 and r^2^ = 0.126 resp.)(Fig 7A and 7B), but it showed a strong positive correlation with the total number of intra-lymphatic leukocytes (r^2^ = 0.443; p = 0,001) (Fig 7C). Serum urea levels also showed a positive correlation with the total number of intra-lymphatic leukocytes (r^2^ = 0.483; p<0,001)(Fig 7F), but not with interstitial inflammation and interstitial fibrosis (r^2^ = 0.007 and r^2^ = 0.003 resp.)(Fig 7D and 7E). The same effect can be seen in the correlation between creatinine clearance and the histological kidney injury parameters. Creatinine clearance showed an inverse correlation with the total number of lymphatic leukocytes (r^2^ = 0.323; p = 0,009)(Fig 7I) and no correlation with interstitial inflammation and interstitial fibrosis (r^2^ = 0.065 and r^2^ = 0.009 resp.)(Fig 7G and 7H).

## Discussion

This study indicates that lymph vessels formed in renal transplants are actively involved in the migration of leukocytes. These migrating leukocytes consist mostly of MHCII^+^, CD103^-^, ED-1^-^ and HIS14^-^ APCs. We show that treatment of transplanted rats with (non-) anticoagulant glycol-split heparin increases the presence of leukocytes in lymphatic vessels, predominantly MHCII^+^, ED-1^-^, CD103^-^ and HIS14^-^ APCs, possibly by tissue diffusion of lymphatic chemokines such as CCL21 from lymphatic HSPGs. And finally we show that lymphatic leukocyte accumulation correlates more with loss of renal function compared to classic histological hallmarks of alloresponse i.e. interstitial inflammation and fibrosis.

It is an ongoing debate whether newly-formed lymph vessels are actively involved in leukocyte migration towards lymph nodes [5]. Our results indicate that the newly formed lymph vessels are active in producing chemokines and attracting leukocytes. Furthermore we show an upregulation of perlecan and podoplanin expression in transplanted kidneys, which might suggest an increase in the formation of lymph vessels and underlying basement membrane which hosts perlecan as one of its proteoglycans. Perlecan, amongst other proteoglycans, acts as a docking platform for lymphatic chemokines like CCL21 and thereby facilitates the formation of a chemotactic gradient for leukocytes in the lymphatic basement membrane. CCL21 is seen as the most important chemokine for lymphatic control of migration [18]. In our study we show a strong CCL21 expression in the tubulo-interstitium of allografted kidneys. This finding is in accordance with literature showing that activated primary human dermal lymphatic endothelial cells secrete and produce CCL21 upon inflammatory stimuli [31]. Interestingly, treatment of allograft kidneys with glycol-split heparin reduces CCL21 staining in the kidney.

The migration of leukocytes towards the lymph vessels is of major importance in the development of an allograft response. In our study we show that lymphatic leukocyte accumulation inversely correlates with renal function, as opposed to classic histological damage parameters like interstitial inflammation and fibrosis [3]. To our knowledge this has not been shown before. The dominant leukocyte subset migrating across lymphatic endothelium in our study is the MHCII^+^, CD103^-^, ED-1^-^ and HIS 14^-^ APC which shows that it is most likely a subset of DC. Classical non-lymphoid DCs are devided in a CD103^+^, CD11b^-^ and a CD103^-^, CD11b^+^ group [32]. The main function of CD103^+^, CD11b^-^ DCs is thought to be cytokine production and cross-presentation [33]. CD103^-^, CD11b^+^ DCs are characterized by their strong expression of MHC class II and this subset of DCs is therefore thought to be important in the induction of CD4^+^ T cells [34,35]. In chronic allograft dysfunction it has been demonstrated before that CD4^+^ T cells play a pivotal role in the onset of remodeling [36]. Since the intralymphatic leukocyte population in our study is MHC class II^+^ while being CD103^-^, ED-1^-^ and HIS 14^-^, these cells probably function as CD4+ T-cell activating APCs and are most likely dendritic cells of the CD103^-^, CD11b^+^ subtype. Studies using anti-CCL21 antibodies have shown that CCL21 is critical in DC migration [13]. PCR data in our model shows a substantial increase in CCL21 expression and staining for CCL21 shows substantial release of CCL21 in the interstitium, without differences in the number of lymph vessel in the tissue. This might be an explanation why MHCII^+^, CD103^-^ ED-1^-^ and HIS14^-^ APCs are most abundantly leukocyte migrating in transplantation.

We could not show a positive effect of heparin/ non-anticoagulant heparin derivatives treatment on graft function and outcome, despite others reported this [20,37,38]. Experiments performed by Braun and colleagues showed that LMWH reviparin can improve renal function and reduce inflammation in experimental renal transplants [20]. We could repeat the reduction of inflammation by treatment with glycol split heparin, but we failed to show improved renal function. This can be explained by the differences in study design, since we treated with high molecular weight heparin/ non-anticoagulant heparin derivatives and used a full HLA mismatch model compared to the milder Lewis to Fisher model used in the studies of Braun et. al. The influence of molecular weight of heparin/ non-anticoagulant heparin derivatives can be the subject of future research. The most prominent difference with former studies is that we show an increase in leukocyte accumulation in lymph vessels by treatment with glycol-split heparin. As far as we know this effect of heparin/ non-anticoagulant heparin derivatives has never been shown before and might well explain the decreased renal function parameters in the glycol-split heparin treated group.

In vitro we showed an inhibitory effect of glycol-split heparin on the binding of CCL2 and CCL21 to immobilized HSPG’s. The effect of glycol-split heparin treatment on leukocyte influx from the vascular system might be explained by ‘elution’ of chemokines such as CCL2 from endothelial proteoglycans, preventing activating and transmigration of rolling leukocytes. This might explain the reduced influx of leukocytes seen in the glycol-split heparin treated group. The stronger affinity of glycol-split heparin (in comparison with unmodified heparin) for CCL2 could be explained by the chain flexibility induced by the presence of glycol split residues that can allow a better interaction with CCL2.

The increased lymphatic accumulation of leukocytes and the higher binding affinity for CCL21 *in vitro* in the glycol-split heparin treated group might indicate that CCL21 can disperse more *in vivo* by binding to glycol-split heparin. This could expand the chemotactic gradient and therefore increase leukocyte migration towards lymph vessels. This is supported by the finding that CCL21 is less abundantly present in the peri-lymphatic area in glycol-split heparin treated animals. The migration stimulatory effects of HS or heparin have been shown before *in vitro* for other heparin binding cytokines and chemokines like CXCL12 (SDF-1) and IL-8 [39,40]. Another aiding factor might be that HS chains can oligomerize CCL21 which might increase the affinity for CCR7 [16]. The oligomerization of CCL21 upon binding with glycol-split heparin might also stimulate the effectiveness of CCL21 to activate interstitial leukocytes, adding to the effect of glycol-split heparin as a CCL21 potentiator. Lately more evidence is accumulating which indicates that HS might act as a co-receptor for chemokine presentation [16,41]. *In vitro* heparin, like glycol-split heparin, also showed a high binding affinity for CCL21 and subsequently heparin treatment of transplanted kidneys showed a high dispersion of CCL21 *in vivo*. Heparin treatment, however, failed to show increased DC accumulation in lymph vessels. This discrepancy might be explained by the fact that glycol splitting of heparin makes it less vulnerable to heparanase cleaving, an abundant enzyme in inflammation [42].

The results in our study lead to the conclusion that heparin/ non-anticoagulant heparin derivative treatment in transplantation is not beneficial for graft function and survival. We do, however, show a novel effect of heparinoids in inflammation. The increase of leukocyte trafficking towards lymph vessel is certainly not preferable in inflammatory conditions like transplantation. However, in conditions in which increased antigen presentation is desired this effect can be beneficial. The trafficking and transmigration of DCs has been of interest in multiple research areas such as vaccine and cancer research [43]. In these research fields it has been shown that *in vivo* stimulation can activate DCs and increase their homing [44]. Since we show this effect for glycol-split heparin, it might be that glycol-split heparin is of interest in these fields. In transplantation the focus of DC research has mainly been on the in vitro propagation of tolerogenic donor and recipient DCs to induce T lymphocyte allograft tolerance [45]. DC homing is of major importance in these therapies so glycol-split heparin could be a valuable addition to these studies as well.
